# Quantitatively detecting *Candida albicans* enolase1 with a one-step double monoclonal antibody sandwich ELISA assay

**DOI:** 10.3389/fmicb.2023.1078709

**Published:** 2023-02-20

**Authors:** Jingzi Piao, Ning Li, Lina Zhang, Hanbing Meng, Qingqing Sun, Zhengxin He

**Affiliations:** ^1^College of Plant Protection, Shenyang Agricultural University, Shenyang, Liaoning, China; ^2^Shenyang Institute for Food and Drug Control, Shenyang, Liaoning, China; ^3^Basic Medicine Laboratory, Bethune International Peace Hospital, Shijiazhuang, Hebei, China

**Keywords:** invasive candidiasis, laboratory diagnosis, *Candida albicans* enolase1, double antibody sandwich enzyme-linked immunosorbent assay, rabbit model

## Abstract

Invasive candidiasis (IC) is often a cause of severe concern for the hospitalized patients, particularly those who are critically sick. However management of this disease is challenging due to a lack of effective laboratory diagnostic techniques. Hence, we have developed a one-step double antibody sandwich enzyme-linked immunosorbent assay (DAS-ELISA) using a pair of specific monoclonal antibodies (mAbs) for the quantitative detection of *Candida albicans* enolase1 (CaEno1), which is considered as an important diagnostic biomarker for IC. The diagnostic efficiency of the DAS-ELISA was evaluated by using a rabbit model of systemic candidiasis and compared with other assays. The method validation results demonstrated that the developed method was sensitive, reliable, and feasible. The findings of the rabbit model plasma analysis indicated that the diagnostic efficiency of the CaEno1 detection assay was better in comparison to the (1,3)-β-D-glucan detection and blood culture. CaEno1 is present in the blood of infected rabbits for a brief period and at relatively low levels and thus the combination of CaEno1 antigen and IgG antibodies detection could aid to increase diagnostic efficiency. However, to improve the clinical application of CaEno1 detection in the future, efforts should be made to increase the detection limit of the test by promoting technical developments and by optimizing the protocol for the clinical serial determinations.

## Introduction

Over the last few decades, the prevalence of nosocomial fungal infections, including invasive candidiasis (IC), has increased significantly. For critically ill patients, IC is frequently associated with high mortality rates and high medical costs (Antinori et al., 2016; Pappas et al., 2018). Epidemiological studies have revealed that five distinct species can cause over 90% of *Candida* invasive infections: *Candida albicans*, *Nakaseomyces (Candida) glabrata*, *Candida tropicalis*, *Candida parapsilosis*, and *Candida krusei (Pichia kudriavzevii)*. *Candida albicans* is the most common cause of bloodstream infections, though infections caused by the non-*albicans* species are becoming more common (Kullberg and Arendrup, 2015).

Early diagnosis and prompt antifungal therapy are critical for markedly improving the outcome of patients with IC (Pappas et al., 2016). However, the lack of robust diagnostic assays remains a major challenge in facilitating an accurate diagnosis of IC. Currently, *Candida* detection techniques used in the clinical laboratories have various fundamental methodological flaws. For example, the microbial cultures suffer from limited sensitivity and long sample turnaround times; (1,3)-β-D-glucan tests have limitations in that they cannot effectively discriminate between the fungal species and are prone to give false positives (Ito et al., 2018; Tschopp et al., 2022).

Nucleic acid detection assays have been found to be more sensitive than the traditional microbial culture and could be used as a complementary diagnostic technique to diagnose IC (Ramos et al., 2017). Although several commercial *Candida* molecular detection systems are available, their clinical utility is largely limited due to a lack of assay standardization and low nucleic acid extraction efficiency (Fuchs et al., 2019; Hayette et al., 2019). T2Candida is a United States Food and Drug Administration (FDA)-approved nanodiagnostic panel that can detect the five most commonly isolated *Candida* species directly from the whole blood in about 5 h (Hamula et al., 2016; Lamoth et al., 2020; Zurl et al., 2020). This robust test has been available for years and is still being refined; yet is often inaccessible to the routine laboratories and the patient populations they serve due to a lack of widespread availability.

Another important strategy for IC diagnosis is the rapid detection of *Candida* antigenic proteins or their antibodies, providing a simple, time-saving, and efficient option. A number of proteins have been identified as the potential diagnostic markers, including the *C. albicans* enolase1 (CaEno1; Li et al., 2013; He et al., 2015, 2016; Pitarch et al., 2018). As early as in 1991, Walsh et al. reported *Candida* enolase antigenemia in cancer patients with IC (Walsh et al., 1991). Despite significant advances in immunological techniques for the laboratory diagnosis in recent years, a mature and stable method for the detection of CaEno1 has yet to be reported. In a previous study, we have generated two CaEno1-specific monoclonal antibodies (mAbs): 9H8 and 10H8, and their specificities were validated using the liquid chromatography with tandem mass spectrometry (LC–MS/MS) and Western blotting (He et al., 2022). Here, we have further developed and tested a one-step double antibody sandwich enzyme-linked immunosorbent assay (DAS-ELISA) for the quantitative detection of CaEno1 antigen using the two mAbs. Furthermore, the diagnostic efficiency of the DAS-ELISA was evaluated by employing a rabbit model of systemic candidiasis and compared with other assays.

## Materials and methods

### Organism and growth conditions

In this study, *C. albicans* strain SC5314 was used (He et al., 2015). *C. tropicalis* ATCC1369, *N. glabrata* ATCC15126, *C. parapsilosis* ATCC22019, and *C. krusei (P. kudriavzevii)* ATCC6258 were obtained from the American Type Culture Collection (ATCC). The cells were subcultured on sabouraud dextrose agar (SDA) and incubated for 24 h at 35°C for all experiments. An inoculum from SDA plates was resuspended in the yeast extract peptone dextrose medium (YPD, 1% yeast extract, 2% peptone, and 2% dextrose) and incubated at 35°C for 48 h. The yeast cells were centrifuged, washed twice with the phosphate buffered saline (PBS; pH7.4, 0.01 M), and densities were determined by plate counting or measuring optical density values at 600 nm (OD600).

### Whole cell ELISA

Whole cell ELISA was used to determine the specificity of mAbs 9H8 and 10H8 across different clinically important pathogens. The production of 9H8 and 10H8 mAbs has been described previously (He et al., 2022).

*Candida albicans* cells were adjusted to an OD600 of 0.5 in a 0.05 M carbonate buffer solution (CBS) that contained 15 mM Na_2_CO_3_ and 35 mM NaHCO_3_ at pH9.6. The prepared suspensions were then used to coat the ELISA plates (100 μl/well) overnight at 4°C. As the negative controls, a panel of clinically relevant organisms including *Escherichia coli*, *Klebsiella pneumoniae*, *Pseudomonas aeruginosa*, *Acinetobacter baumannii*, *Enterococcus faecium*, *Enterococcus faecalis*, *Streptococcus pneumoniae*, *Staphylococcus aureus*, *Staphylococcus epidermis*, *Cryptococcus neoformans*, *C. tropicalis*, *N. glabrata*, *C. parapsilosis*, *C. krusei (P. kudriavzevii)*, *Saccharomyces cerevisiae*, and *Candida dublinensis* were used. These strains were grown, resuspended in CBS (OD600 = 0.5) and then coated onto ELISA plates. The plates were washed three times with PBS containing 0.05% Tween 20 (PBST), blocked with 100 μl of PBST plus 3% (w/v) bovine serum albumin (BSA), and incubated for 2 h at 37°C. The plates were treated for 1 h at 37°C with 10H8 or 9H8 that were diluted to 1 μg/ml by PBST plus 1% (w/v) BSA, followed by addition of 1:5,000 diluted horseradish peroxidase conjugated (HRP) goat anti-mouse IgG (Solarbio, Beijing China). After washing, 100 μl of the 3,3′,5,5′-tetramethylbenzedine (TMB) solution (Beyotime, Shanghai, China) was added to each well and incubated at 37°C for 10 min. The reaction was stopped with 50 μl of 2 N H_2_SO_4_, and the OD450 values were finally measured with the VersaMax plate reader (Molecular Devices, CA, United States).

### The one-step DAS-ELISA

The recombinant CaEno1 was used as a standard (He et al., 2015). The ELISA assay uses the Sandwich-ELISA principle and checker-board analyses were performed to determine the optimal concentrations of both the capture and detection antibodies.

**Figure 1:**
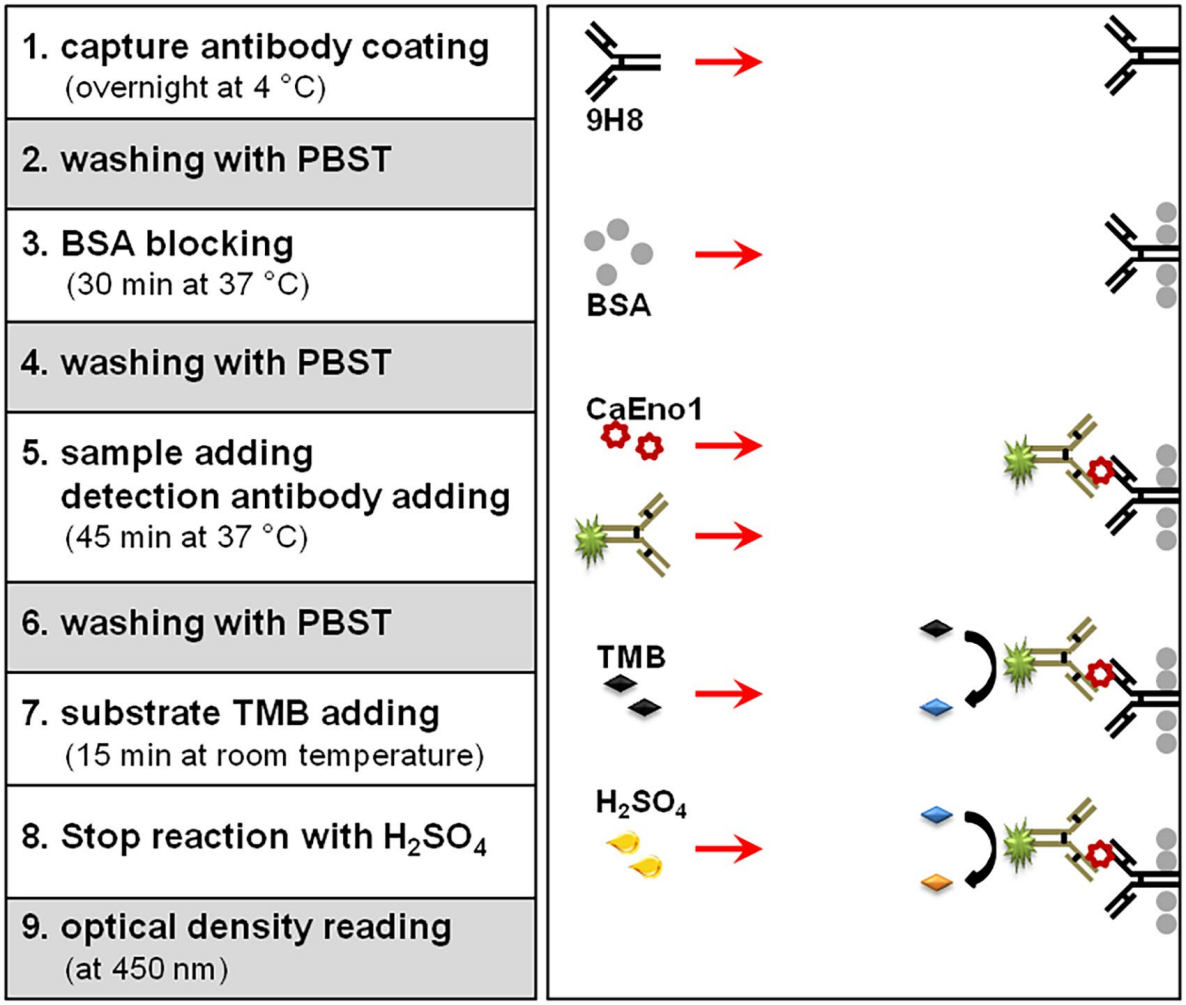
Schematic representation of the one-step double monoclonal antibody sandwich ELISA assay.

Figure 1 depicts the protocol scheme for the DAS-ELISA. Briefly, mAb 9H8 (3 μg/ml in CBS, pH 9.6, 100 μl) was coated overnight at 4°C in each well of a 96-well microplate. After washing trice with PBST, the wells were blocked with 100 μl of PBST plus 3% (w/v) BSA, and incubated for 30 min at 37°C. The plasma samples (or diluted standards, 50 μl/well) and 50 μl of an optimized dilution (1:2,000) of HRP-10H8 were thereafter added to the wells and incubated at 37°C for 45 min. After washing, TMB substrate was applied to each well and incubated for 15 min at room temperature. The reaction was terminated, and the OD450 values were measured. The results were analyzed by using the software of ELISACalc V0.2.

The calibration curves were generated by calculating the ratios between the OD450 values and the corresponding standard concentration. The linearity was determined by serial dilution of a mock plasma sample with high CaEno1 concentration. The limitation of detection (LOD) was calculated by signal blank ± 3SD (Dixit et al., 2011). The recovery tests were conducted to evaluate the accuracy of the DAS-ELISA (Shen et al., 2020).

### Rabbit model of systemic candidiasis

Three female New Zealand white rabbits weighing approximately 2.5 kg were procured from Hebei Medical University’s Experimental Animal Center (Shijiazhuang, China). The Bethune International Peace Hospital’s Ethics Committee authorized the animal care and use methods (No.2019-KY-23), and all the applicable institutional and governmental standards regulating the ethical use of animals were followed.

Each rabbit was inoculated with 1.5 × 10^6^ CFU of *C. albicans* SC5314 blastospores suspended in PBS intravenously through the marginal ear vein to cause the systemic candidiasis. Both the body temperature and weight of the rabbits were measured before the inoculation and on the 1st, 2nd, 4th, 7th, 10th, 15th, 20th, and 27th days post inoculation. Approximately 3.0 ml of anticoagulant blood was obtained from the model rabbits at each time point for different tests. Among them, 100 μl of whole blood was used for the blood hematology that was carried out with Sysmex 2100 (Sysmex Corporation, Kobe, Japan). The platelet rich plasma was obtained from 400 μl of blood and used to analyze the (1,3)-β-D-glucan concentration with a commercial kit by using the kinetic turbidimetry method (Xiamen Bioendo Technology Co., Ltd., Xiamen, China). The remaining 2.5 ml of blood was thereafter centrifuged to separate the plasma and blood cells. The plasma was utilized for biochemical analysis, as well as the detection of CaEno1 antigen and antibodies. Blood biochemistry analysis was performed by an Olympus AU5400 system (Olympus, Shinjuku, Japan). Six biochemical tests were used to assess the liver, kidney, and heart function of the rabbits: total protein (TP), alanine aminotransferase (ALT), aspartate aminotransferase (AST), plasma blood urea nitrogen (BUN), and phosphocreatine kinase (CK). The residual blood cells were resuspended in the normal saline to 2.5 ml and injected into the children’s blood culture bottles, where they were cultivated by using the BacT/ALERT 3D automated system (bioM é rieux, Craponne, France). The positive samples were subcultured on the standard media by using the routine microbiological techniques.

### CaEno IgG antibody detection

*Candida albicans* enolase1 IgG antibody in rabbit plasma was measured by indirect ELISA, as previously described (He et al., 2015). Recombinant CaEno1 was then coated onto each well of blank ELISA plates for overnight at 4°C after being diluted to 1 μg/ml in CBS. After washing trice with PBST, the plates were then blocked with PBST plus 3% (w/v) BSA solution, and incubated for 2 h at 37°C. After washing, rabbit plasma samples with a 1:500 dilution by PBST plus 1% (w/v) BSA were added and incubated at 37°C for 1 h. After three washes with PBST, 1:5,000 diluted HRP-conjugated goat anti-mouse IgG (Solarbio, Beijing, China) was added and incubated for 1 h at 37°C. After another round of washing, 100 μl of TMB substrate solution was applied to each well and incubated for 10 min at 37°C. Thereafter, the reaction was terminated and the OD450 values were determined.

## Results

### Specificity of the mAbs and DAS-ELISA

We have previously demonstrated that 9H8 and 10H8 can specifically recognize CaEno1 in total lysate from *C. albicans*. We have also developed whole-cell ELISA assays to determine the response specificity of the 9H8, 10H8 mAbs against the different clinically important pathogens including a panel of *Candida* spp. and non-*Candida* organisms. The specificity of the DAS-ELISA was determined by detecting CaEno1 in the supernatant of various *Candida* spp. and grown for 48 h in YPD medium. The other negative control organisms were grown in enrichment broth and the supernatant was applied to the DAS-ELISA test as well. Table 1 depicts that 9H8 mAb can react specifically with *C. albicans*, with no cross interaction with other organisms that were evaluated. Remarkably, 10H8 mAb exhibited distinct response characteristics and could cross-react with all the *Candida* spp. and *S. cerevisiae*. The two mAbs’ DAS-ELISA could specifically react with the *C. albicans* culture supernatant.

**Table 1:** Reaction specificity of 9H8, 10H8 mAbs, and the DAS-ELISA.

Species	Source of strain^a^	Cross reactivity		
		9H8	10H8	DAS-ELISA^b^
*Candida albicans* strain SC5314	Standard strain	Positive	Positive	Positive
*Candida tropicalis* ATCC1369	Standard strain	Negative	Positive	Negative
*Nakaseomyces (Candida) glabrata* ATCC15126	Standard strain	Negative	Positive	Negative
*Candida parapsilosis* ATCC22019	Standard strain	Negative	Positive	Negative
*Candida krusei (Pichia kudriavzevii)* ATCC6258	Standard strain	Negative	Positive	Negative
*Saccharomyces cerevisiae* ATCC9763	Standard strain	Negative	Positive	Negative
*Candida dublinensis* (*n* = 3)	Clinical isolates	Negative	Positive	Negative
*Cryptococcus neoformans* (*n* = 3)	Clinical isolates	Negative	Negative	Negative
*Escherichia coli* (*n* = 3)	Clinical isolates	Negative	Negative	Negative
*Klebsiella pneumoniae* (*n* = 3)	Clinical isolates	Negative	Negative	Negative
*Pseudomonas aeruginosa* (*n* = 3)	Clinical isolates	Negative	Negative	Negative
*Acinetobacter baumannii* (*n* = 3)	Clinical isolates	Negative	Negative	Negative
*Enterococcus faecium* (*n* = 3)	Clinical isolates	Negative	Negative	Negative
*Enterococcus faecalis* (*n* = 3)	Clinical isolates	Negative	Negative	Negative
*Streptococcus pneumoniae* (*n* = 3)	Clinical isolates	Negative	Negative	Negative
*Staphylococcus aureus* (*n* = 3)	Clinical isolates	Negative	Negative	Negative
*Staphylococcus epidermis* (*n* = 3)	Clinical isolates	Negative	Negative	Negative

a All the clinical isolates have been identified by an ISO9000 certified microbiological clinical laboratory.

b DAS-ELISA, dual antibody sandwich enzyme-linked immunosorbent assay.

### Performance of the DAS-ELISA

**Figure 2:**
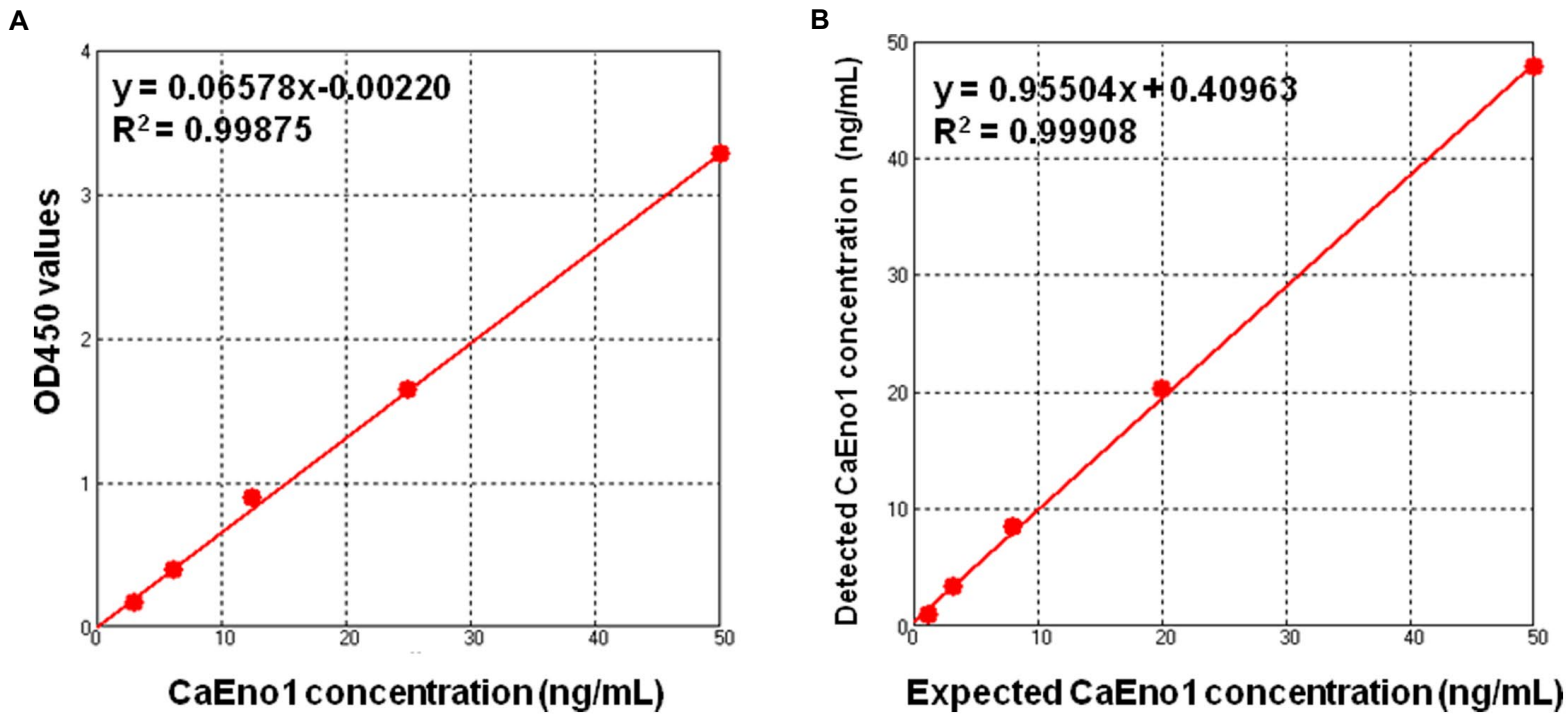
Standard curve **(A)** and linear range **(B)** of the one-step DAS-ELISA assay for the quantitative detection of CaEno1 in rabbit plasma.

Figure 2A depicts a standard curve generated with the recombinant CaEno1 as the standard. Normal rabbit plasma serial dilution of the recombinant CaEno1 was used as mock clinical samples, and the result indicated good linearity of DAS-ELISA across the range that we have investigated (top limit was close to 50 ng/ml, bottom limit was about 1 ng/ml, Figure 2B). The LOD of CaEno1 detection by this method was 0.33 ng/ml. The recovery test was conducted by addition of a high value standard sample into a low value matrix sample, with the volume ratio of 1:9. The recovery rate was determined by using the following formula: Recovery (in %) = [(amount detected − amount sample)/amount standard spiked] × 100. The recovery experiment was repeated trice, with an average recovery rate of 103.26% (95% CI = 85–115%).

### Rabbit model of the systemic candidiasis

**Figure 3:**
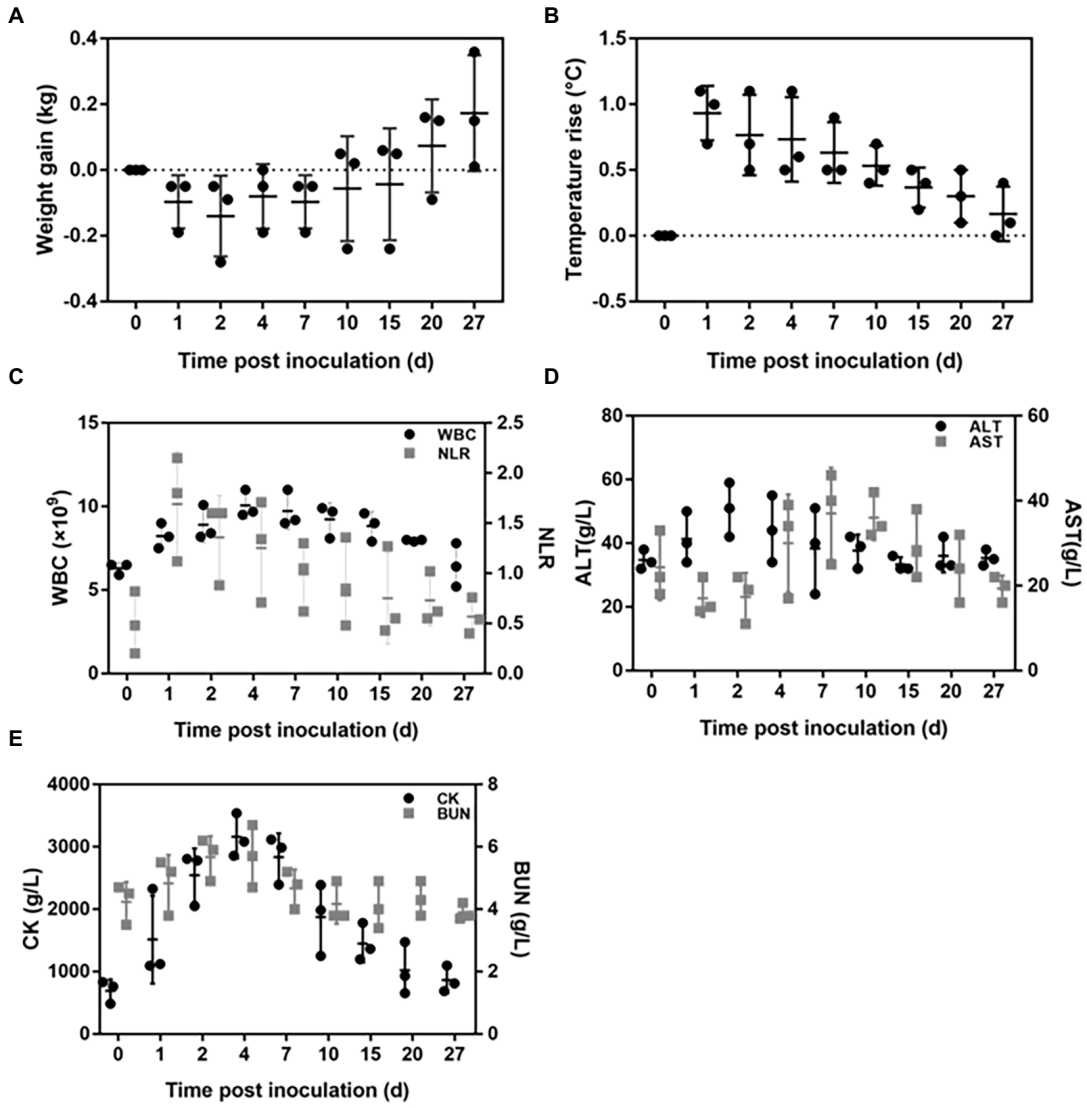
Dynamic changes in both the physiological and biochemical indicators after *Candida albicans* SC5314 inoculation in rabbits. **(A)** Body weight; **(B)** Body temperature; **(C)** White blood cell (WBC) count and neutrophil lymphocyte ratio (NLR); **(D)** Plasma alanine aminotransferase (ALT) and aspartate aminotransferase (AST); **(E)** Plasma blood urea nitrogen (BUN), and phosphocreatine kinase (CK).

All the rabbits displayed infection symptoms after being inoculated with *C. albicans* SC5314, such as loss of appetite and reduced activity. The body weight decreased significantly after infection (Figure 3A), and the body temperature increased dramatically on the first day, then gradually recovered to normal (Figure 3B). The number of peripheral white blood cells (WBC) rose steadily beginning on the first day after the injection and peaked on the seventh day. The dynamics of the neutrophil lymphocyte ratio (NLR) were remarkably comparable to the dynamics of the body temperature. It increased rapidly on the first day and then was gradually restored to the normal value (Figure 3C). The biochemical tests showed that once rabbits were infected with *C. albicans*, the heart, liver, and kidney functions were impaired. The peak of ALT emerged on the second day after *Candida* inoculation, the peak of AST level was reached on the seventh day, whereas the peaks of both CK and BUN level appeared on the fourth day (Figures 3D,E).

### Diagnostic efficiency of CaEno1 detection was better in comparison to the other tests

**Figure 4:**
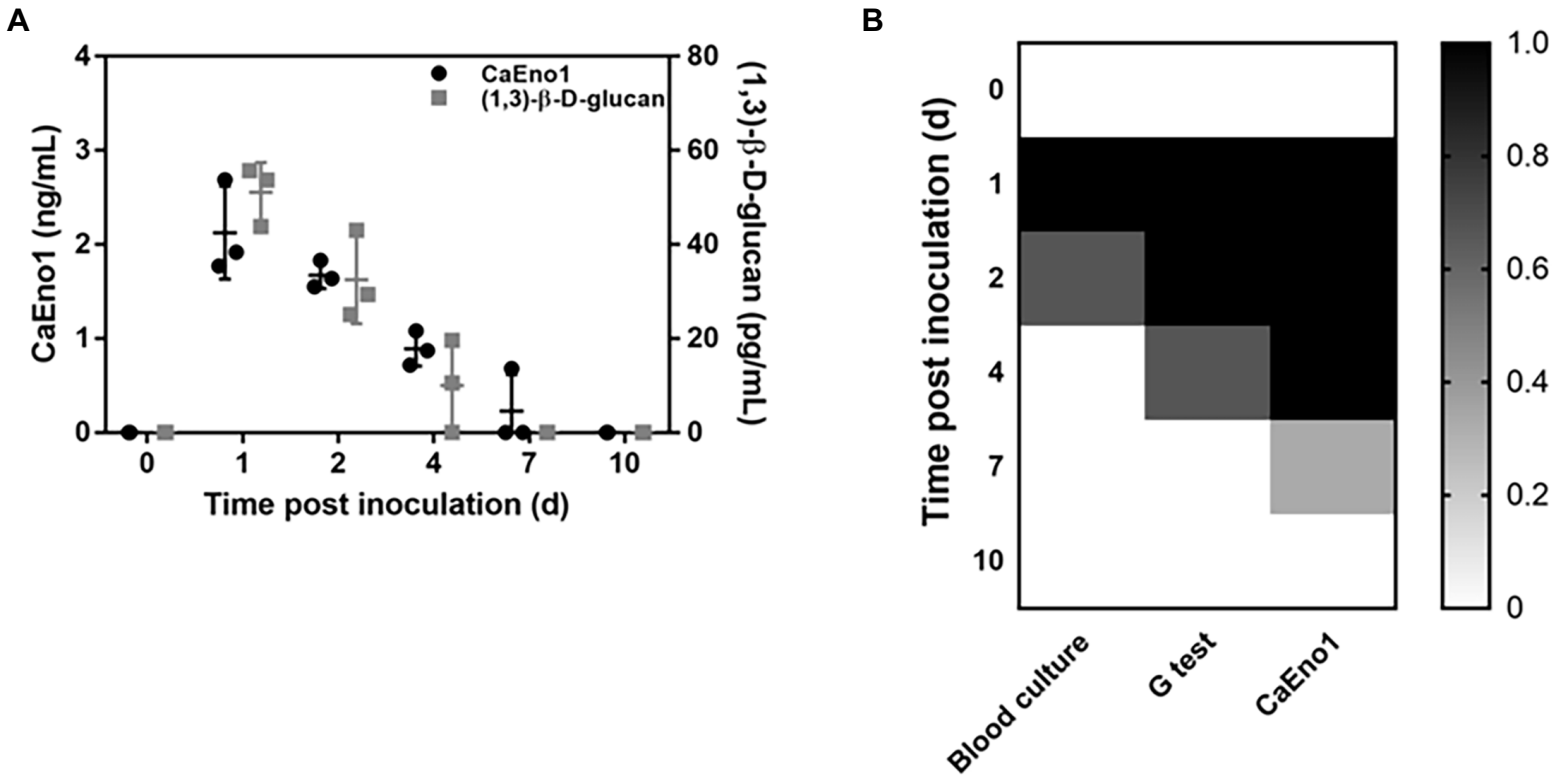
Dynamics of CaEno1 and its diagnostic efficiency in comparison to the *G*-test and blood culture. **(A)** Dynamics of CaEno1 and (1,3)-β-D-glucan (cut-off, 10 pg./ml) in rabbit blood after *Candida albicans* inoculation; **(B)** Diagnostic efficiency of CaEno1 detection, *G*-test, and the blood culture.

*Candida albicans* enolase1 and (1,3)-β-D-glucan were analyzed in rabbit plasma samples, and their dynamics have been depicted in Figure 4A. CaEno1 and (1,3)-β-D-glucan were found in the peripheral blood of rabbit following fungal inoculation. Both the levels in the blood steadily declined over time. On the seventh day after inoculation, (1,3)-β-D-glucan could not be detected in the rabbit model’s blood, and only one rabbit assessed positive for CaEno1. The blood culture findings revealed that all the rabbits had positive results after fungal inoculation. One rabbit remained positive for 24 h, whereas the two others remained positive for 48 h. In addition, upon microscopic analysis, blastospores and hyphal *Candida* could be observed in all blood positive cultures that were recognized as *C. albicans* by CHROMagar.

We next compared the diagnostic efficiency CaEno1, *G*-test, and blood culture. Each rabbit was assigned a value of 1 for each positive result and a value of 0 for each negative result, yielding the heat map as displayed in Figure 4B. On the first day after inoculation, all the tests performed identically to diagnose IC; on the second day, the CaEno1 detection and the *G*-test still diagnosed all rabbits, and better than the blood cultures; on the fourth and seventh days, CaEno1 detection diagnostic efficiency was slightly better than the *G*-test, while the blood culture diagnostic efficiency was 0.

### Combined CaEno1 antigen antibody detection improved the diagnostic efficiency

**Figure 5:**
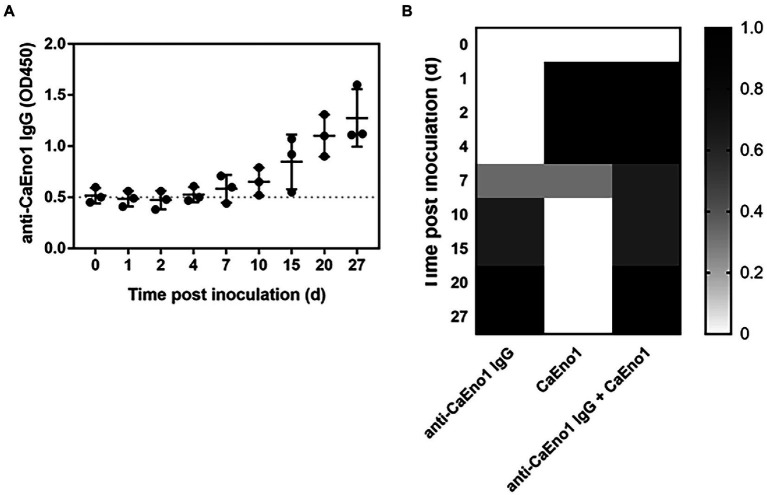
Combined CaEno1 antigen–antibody detection enhanced diagnostic efficiency in rabbits inoculated with *C.albicans*. **(A)** Anti-CaEno1 IgG dynamics in rabbit plasma; **(B)** Diagnostic efficiency of anti-CaEno1 IgG, CaEno1 detection, and their combined application.

All rabbits generated anti-CaEno1 IgG after being inoculated with *C. albicans*. The antibodies began to appear around day 7 after inoculation and steadily rose subsequently, and continued to grow until the experiment ended on day 27 (Figure 5A).

Indirect ELISA exhibited a significant background when detecting anti-CaEno1 IgG antibodies in rabbit plasma samples. We used rabbit plasma before inoculation as a control and tested it 20 times, with the OD450 > mean OD450_control_ + 2SD_control_ set as the threshold for determining positive samples. The heat map in Figure 5B was produced by assigning a value of one to each positive result and a value of zero to each negative result for each rabbit. The results demonstrated that the CaEno1 antigen and antibody test exhibited high complementarily for the diagnosis of *Candida* infection. The positive antigen findings were mostly found in the initial stages, whereas the positive antibody results were mostly observed in the late stages of infection. The combined antigen–antibody tests resulted in the successful diagnosis in all rabbits from days 1 to 4 and 20 to 27 after *Candida* inoculation, as well as in a portion of rabbits from days 4 to 15.

## Discussion

Since IC is subtle and indistinguishable from the bacterial infections, it presents a major challenge in clinical diagnosis. However, in recent years, researchers have investigated a combination of various culture and non-culture techniques, as well as tested the combined use of different non-culture techniques, for enhancing the diagnostic efficiency (Posteraro et al., 2011; Barantsevich and Barantsevich, 2022; Fisher et al., 2022). At the same time, novel diagnostic technologies are constantly being developed and clinically tested. Here, we have developed a one-step double antibody sandwich ELISA for CaEno1 detection by using a pair of previously generated mAbs (He et al., 2022), lowering the detection time to less than 2 h. Conversely, it takes 2–5 days for the culture procedure to achieve conclusive results (Pitarch et al., 2018). The long sample turnaround time of the culture frequently postpones the optimal time for IC management. Furthermore, the ELISA test is easy to perform in the routine labs. Since few extremely sensitive diagnostic techniques, such as T2Candida, are not widely available, the IC diagnostic technique based on ELISA could be used as a complement to them.

Antigen testing is a powerful technique for screening and diagnosing the different infectious diseases, and it has been found to play a crucial role in the management and control of the infectious diseases such as the COVID-19 epidemic (Charton et al., 2020; Ciotti et al., 2021; Peeling et al., 2021). When compared to other commonly employed techniques such as culture and nucleic acid testing, antigen testing is faster and less expensive, thus allowing for larger-scale screening and dynamic surveillance. Despite the fact that a variety of *Candida* proteins, such as Eno1 (Walsh et al., 1991), Sap1/2(Shukla et al., 2021), and Mp65 (Gomez et al., 2000), have been identified as potential biomarkers for the diagnosis of IC, commercial diagnostic kits for the detection of *Candida* antigens are scarce. CAND-TEC™ is an approved kit for the latex agglutination detection of unknown heat-stable *Candida* proteins; nevertheless, the test has a limited diagnostic sensitivity for IC (Held et al., 2013). CaEno1 is a highly conserved protein that *C. albicans* actively secretes or releases exclusively during the process of invasive infections (Sundstrom and Aliaga, 1992). Since commercial kits for CaEno1 detection have been seldom reported, development of innovative one-step DAS-ELISA that can detect CaEno1 at concentrations over 0.33 ng/ml has the potential to be converted into a commercial kit once the detection technology has been enhanced and optimized.

A rabbit model of systemic candidiasis was established to assess the ability of DAS-ELISA to detect CaEno1 and diagnose IC. The plasma samples obtained from infected rabbit models were analyzed using the one-step DAS-ELISA, and the concentrations of CaEno1 and (1,3)-β-D-glucan decreased as the infection time was increased. The fundamental explanation, we believe, could be attributed to the fact that immunocompetent rabbits have a high resistance to *Candida* infection, and the injected *C. albicans* is rapidly cleared from the bloodstream. In the clinical circumstances, however, especially in the critically sick patients with compromised immune systems, the host-*Candida* pathogen conflict may be prolonged. For example, the findings from an epidemiological study in the neonatal ward has indicated that the median duration of candidaemia in neonates was 6 days and that 52% of neonates with *Candida* bloodstream infections suffered from persistent candidaemia (Levy et al., 2006). Therefore, it is clinically possible that the CaEno1 antigen in immune-compromise IC patients could continue to remain at an elevated level, thereby rending the detection of CaEno1 more convenient and valuable.

The detection of CaEno1 has distinct advantages over the other diagnostic techniques. It can specifically confirm *C. albicans* infection in comparison to the *G*-test and is simpler, faster, and more sensitive when compared to the blood cultures. In addition, various prior studies have confirmed that CaEno1 IgG antibody detection can play a significant role in IC diagnosis (Philip et al., 2005; Laín et al., 2007; Clancy et al., 2008; Li et al., 2013; Pitarch et al., 2014; He et al., 2015, 2016). Antigen–antibody combination testing has also been shown to increase the diagnostic efficiency in infections caused by several organisms (Mikulska et al., 2010; Fry et al., 2011; Bloch et al., 2018; Veyrenche et al., 2021). Furthermore, analyses of the various physiological and biochemical markers revealed that the inflammatory response cycle in the rabbit infection model lasted around 7 days, with detectable CaEno1 in the plasma lasting 4–7 days and anti-CaEno1 IgG antibodies appearing substantially on day 7. In the rabbit *Candida* infection model, CaEno1 antigen and antibody detection can provide complementary advantages and successfully boost the diagnostic efficiency. It is important to point out that the disadvantages associated with indirect ELISA detection of CaEno1 IgG antibodies include presence of high background and the necessity to dilute the samples, both of which need to be addressed in the future.

The study’s conclusions should be viewed considering some limitations. First, we have used preclinical model to illustrate the importance of CaEno1 antigen detection in the diagnosis of IC, and the full application of these findings in humans will need to be validated in future research. Second, this study only includes the data obtained from a small number of animals. Although we have objectively demonstrated the dynamic changes in the physiological and biochemical markers, CaEno1 and (1,3)-β-D-glucan in model animals, the small number of animals preclude the use of robust statistical analysis in this work.

The one-step DAS-ELISA assay was able to trace amounts of CaEno1 (ng level) in the plasma of the rabbit model, which was only slightly above the LOD of the method, thus suggesting that the sensitivity of this method needs further improvement. Furthermore, CaEno1 detection might be impeded, as with the other protein antigens, by rapid clearance from the bloodstream, the formation of immunological complexes with the associated antibodies, and existence of low amount in circulation (Ellepola and Morrison, 2005). In the future, robust technical advancements will allow us to solve some of these limitations by improving the LOD of the test technique and optimizing the protocol for the clinical serial determinations.

## Data availability statement

The raw data supporting the conclusions of this article will be made available by the authors, without undue reservation.

## Ethics statement

The animal study was reviewed and approved by The Bethune International Peace Hospital’s Ethics Committee.

## Author contributions

JP: conceptualization, methodology, formal analysis, and writing—original draft. NL: data curation and writing—original draft. LZ: funding acquisition and resources. HM and QS: validation and writing—review and editing. ZH: conceptualization, funding acquisition, resources, supervision, and writing—review and editing. All authors contributed to the article and approved the submitted version.

## Funding

This work was supported by grants C2021505001 from the Natural Science Foundation of Hebei province, China, grants 20200242 from the Medical Science Research Project of Hebei Province, China, and grants 201200623 from the Science and Technology Research and Development Plan of Shijiazhuang, China.

## Conflict of interest

The authors declare that the research was conducted in the absence of any commercial or financial relationships that could be construed as a potential conflict of interest.

## Publisher’s note

All claims expressed in this article are solely those of the authors and do not necessarily represent those of their affiliated organizations, or those of the publisher, the editors and the reviewers. Any product that may be evaluated in this article, or claim that may be made by its manufacturer, is not guaranteed or endorsed by the publisher.
